# Analysing intersecting social resources in young people’s ability to suggest safer sex - results from a national population-based survey in Sweden

**DOI:** 10.1186/s12889-022-13672-1

**Published:** 2022-07-04

**Authors:** Anna ChuChu Schindele, Kristina Areskoug Josefsson, Malin Lindroth

**Affiliations:** 1 Centre for Sexology and Sexuality Studies, Department of Social Work, Faculty for Health and Society, Malmö University, Malmö, Sweden; 2 Unit for Sexual Health and HIV Prevention, Department of Communicable Disease Control and Health Protection, The Public Health Agency of Sweden, Solna, Sweden; 3 Faculty of Health Studies, VID Specialized University, Sandnes, Norway; 4 Department of Behavioural Science, Oslo Metropolitan University, Oslo, Norway

**Keywords:** Intersectionality, Health promotion, Sexual health, Safer sex, SRHR, Young people

## Abstract

**Background:**

Safer sex is one of the most crucial areas in sexual and reproductive health and rights (SRHR). Drawing on the theory of health promotion where social life generates resources for health our hypothesis is that having control over one’s life situation, affects the ability for safer sex and thereby sexual health. The aim is to explore the association between having control over one’s life and the ability to suggest safer sex among young people aged 16–29, and how this plays out in relation to membership of six constructed social groups based on: gender, transgender experience, sexual identity, economy, being foreign-born, and social welfare recipiency followed by an in-depth analysis of the intersection of gender and sexual identity.

**Methods:**

The data set comprises cross-sectional survey responses from a stratified random sample of 7755 in the total Swedish population of young people. The SRHR-focused questionnaire was developed within the HIV-monitoring program at the Public Health Agency of Sweden. Data collection was conducted by Statistics Sweden between April 15 and June 8 in 2015. The survey had a response rate of 26%, which was in line with the study design. Statistical analysis was used to explore the self-reported outcome variable ability for safer sex and the exposure variable control over one’s life. The methods used comprise multivariate logistic regression and an intersecting multivariate regression exploring 12 intersecting social positions by gender and sexual identity.

**Results:**

The results show that young people’s control over their lives is associated with their ability for safer sex. Due to this, control over one’s life can be seen as a resource for safer sex. The associations in the 12 intersecting social positions showed complex patterns.

**Conclusions:**

The intersections of resources show the complexity and that gender cannot account for all differences in the resources for young people’s ability to suggest safer sex. Implications for policy and practitioners involve both addressing and strengthening the sexual rights of young people from sexual minorities and tailoring interventions in a way that takes the intersections between gender and sexual identity into consideration.

## Background

Sexual and reproductive health and rights (SRHR) are fundamental to health and well-being [1–4]. SRHR includes both social and physical aspects of sexuality and health. Although SRHR covers a broad area of health-related dimensions and outcomes, safer sex is one of the most crucial areas for achieving the highest attainable health [2, 5, 6]. Safer sex enables the WHO vision of sexual health since it promotes pleasurable and safe sexual experiences and prevents sexually transmitted infections (STI), HIV and unintended pregnancies [7]. The promotion of safer includes the potential to empower and protect bodily integrity and make informed and self-made decisions [2, 4]. The ability to achieve the highest attainable sexual health is closely linked to social life and the context in which people live [8, 9]. Moreover, the global research committee of Guttmacher-Lancet concluded that research and practice on safer sex is an area that specifically needs to include socially vulnerable groups such as young people and sexual minorities who often have limited resources to define and control their lives [2, 10]. Whit this background our hypothesis is that feeling able, i.e. ability, to suggest and thus start to negotiate safer sex, is one aspect of life that young people may consider when self-assessing how in control they feel. The hypothesis is also that the intersections of identities within and between social groups affects ability to suggest safer sex. To examine the field, we have used self-reported data from a cross-sectional survey, denoted UngKAB15 (Young people’s Knowledge, Attitudes and Behaviour) in order to learn about the respondents’ subjective thoughts on control over life and ability to safer sex. By doing so we hope to contribute with valuable information about young people’s self-reported and subjective thoughts.

### Being in control of one’s life

One of the domains in the Swedish National Policy on Public Health is “Control over life resources and inclusion in social life”. This domain strives to identify the ways in which human rights and inclusive democracy on a societal level generate empowerment and the ability to have control over one’s life on an individual level and thus can help to improve health [11]. The domain emerges from the social determinants “Social inclusion and non-discrimination” and “Structural conflict” stated by the WHO [12]. Having control over one’s own life situation can be defined as a social resource that affects health, and it is closely linked to how we perceive our social group and our social position in life relative to others. The position relative to others is called the gradient and can be defined as a ladder on which social groups are positioned by social privileges or disadvantages associated with the group [13–15]. Having control over one’s life is a resource that is associated with the potential to experience self-confidence and self-efficacy. Conversely, experiencing a lack of control over one’s life situation can generate a sense of social stress, powerlessness, exclusion, discrimination can result in lower potential enhance social life relative to others [14, 16–18]. The resources needed to be in control of one’s life, and to make autonomous decisions concerning sexuality, are also a core part of health promotion and of the social determinants of health [19, 20]. As a result, health promotion in general, and sexual health promotion in particular, strive to change socio-political factors, often challenging norms in society [16, 21].

### Ability to suggest safer sex

In this context, ability can be defined as the inner individual dimension or capacity to transform social resources in society into health. Ability is closely linked to, and also overlapping with the concepts of empowerment and capability since it situates a person’s prerequisites to act within the complex and mutual construction of an individual person’s social life situation [22–24]. More precisely, ability involves the executive action of a behaviour that can create health within a specific social context. Ability is therefore only as solid as the action (i.e. behaviour) it is supposed to enable [25, 26]. Ability is also dependent on a person’s social position relative to others, and on societal norms and values [27]. From a health promotion perspective, the ability to control social life is a determinant of health outcomes [16]. The ability to suggest safer sex is also closely linked to sexual rights, and requires a non-discriminating society in which young people with a same-sex sexual identity, disabilities, transgender experiences and intersex experience can enjoy pleasurable sexual experiences without being stigmatized, discriminated against or criminalized [28]. However, it is evident that sexual rights are often not respected, and that discrimination and unequal prerequisites create vulnerabilities in relation to sexual health [10, 15, 29–32].

### Intersections

Like in other health areas, sexual health is often analysed through gender, education and income [9, 33, 34]. However, a large number of studies in the field of HIV, for example, show that sexual identity and transgender experience and being born abroad are significant determinants that affect sexual health [35–37]. In order to deepen the analyses and show the complexity, it is also important to analyse the intersections between and within these determinants [38, 39]. Gender, education and income [9, 19, 34] cannot entirely account for variations in men’s, women’s, and gender-diverse people’s access to health services and sexual health outcomes, nor are sex and gender always the most relevant axes for social categorisation [19]. The theory of intersectionality strives to illuminate exclusion and marginalisation, and stipulates that gender needs to be complemented by other social determinants such as sexual identity, ethnicity, migration experiences and economic status [40, 41]. By using intersectionality, it becomes possible to learn how more complex aspects of social life intersect and mutually construct social groups and social position with various resources for the ability to safer sex [42, 43].

### Previous research on the ability for safer sex

The ability for safer sex has mainly been studied by scholars in behavioural science and psychology [44–46]. Bandura developed the field with studies of self-efficacy, in which ability was understood as the possibility for individuals to execute their own desired behaviour within a given social context. The ability for safer sex is often viewed as equivalent to the concepts of sexual self-efficacy and condom self-efficacy [45]. However, as the HIV-pandemic spread in the 1980s and 1990s, research on safer sex became more focused on epidemiology, and shifted its focus to individual risk-taking behaviour [19, 47]. Nevertheless, as early as 1998 the individual risk perspective was criticized for over-emphasizing the role of rational decision-making in sexual behaviour and for ignoring the social and cultural context of human interaction [48]. There is also research based on the public health perspective, which underscores the complexity of safer sex practices among youth. One example is a study from the Bahamas that found that safer sex through condom use among young people was dependent on their social situation, and that the ability for safer sex followed a nonlinear model, which underscores the finding that young people’s safer sex practices are complex. As a result of this nonlinearity, research and interventions need to focus on social determinants and social prerequisites [49]. Moreover, collective gender roles and societal social norms affect young people’s agency and their decision-making ability in relation to safer sex [50–52]. For example, Closson et al. [50] noted that young women reported higher condom self-efficacy and ability than their male peers. However, the young women also reported that they could not buy or bring condoms with them. This indicates that the condom self-efficacy, and thus ability to safer sex, is generated in relation social life [50–52]. Also, findings from a systematic review focusing on behavioural interventions for increased condom use among young people in the US showed that if interventions were based on a theory of behaviour change, in which ability and self-efficacy were central, young people reported significantly increased condom use. Moreover, interventions that were situated in social life, and that promoted methods focused on how to negotiate condom use, and how to get or buy condoms, resulted in both an increased intention to use condoms and increased condom use [53].

Taken together, previous research shows that social life and gender influence the ability for safer sex, and that this ability is linked to gendered power structures and prerequisites for safer sex and sexual health. However, most of the literature is both heteronormative and cis-normative, and little is known about power structures other than the binary gender categories male and female. Thus, safer sex needs to be explored and understood in relation to how gender structures intersect with other social determinants and power structures.

### Aim

The aim is to explore the association between having control over one’s life and the ability to suggest safer sex among young people aged 16–29 in Sweden, and how this plays out in relation to membership of six constructed social groups based on: gender, transgender experience, sexual identity, economy, being foreign-born, and social welfare recipiency followed by an in-depth analysis of the intersection of gender and sexual identity.

## Methods

### Study design

This study and its design emerges from the monitoring program on HIV and SRHR at the Public Health Agency of Sweden [54]. The survey is based on national policies informed by theories of health promotion and the theories of the social determinants of health [16, 55]. The questionnaire items are formulated to learn about barriers to health within the monitoring system of the national program on SRHR and HIV. The target group, young people 16–29 years old, represents the key population defined in national strategies for HIV and SRHR [54, 56]. The survey also monitors domain 7 in the national Public Health Policy “Control and influence on social life“, where resources are mentioned as important prerequisites for health. The questions are formulated for a Swedish context but could be applicable in other countries. They differ from more risk-oriented questions that are often used in epidemiology. However, it can be of importance to note that even more “classical” data such as number of sex partners and condom use are also often self-reported data. With this survey the Public Health Agency also wanted to contribute with an understanding of resources for health in line with the theory of health promotion and the social determinants of health.

### Data collection

The data are based on a cross-sectional survey of a stratified random sample of the total Swedish population of young people. Stratification was made on male of female and design weights were used to weights the responses due to gender. Data was collected by the survey unit at Statistics Sweden (SCB) in accordance with the quality requirements of ISO 20252:2012 for market, opinion, and social surveys. Statistics Sweden’s total population register was used as the sampling frame and gave a sample size of 29,997.

The data collection was done between April 8 until June 8, 2015. The 7755 who completed the survey gives a response rate of 26%. This is line with the power estimations presented in the study design and other Swedish population-based health surveys among youth and young adults [57, 58]. Based on the register of the total population, a non-response analysis was conducted to investigate patterns among those who did not respond to the survey. The non-participants were mainly young men, migrants, and people with low levels of education. The information obtained about the non-respondents was used to calibrate design weights to reduce the impact of the non-response. The data represent the likelihood of estimates for the total population [57] and the results (percentages) are thus considered representative of young people aged 16–29 in Sweden.

### Questionnaire

The survey questions were compiled by Sweden’s Public Health Agency. Following that, the survey questions were examined by Statistics Sweden’s unit for measurement procedures, which put the survey through psychometric test with young people 16–29 years old. The overall report from the piloting session was positive, and the group found the questionnaire interesting and relevant. Only a few changes were made following the piloting session, like clarifying text in the various topics or questions, for example if in a specific question sex were supposed to mean self-sex or sex with a partner [59]. The questionnaire comprised 64 items and covered several perspectives on social life in relation to SRHR and HIV-prevention. To avoid reducing gender to a binary male-female understanding, the questionnaire included non-binary gender as an alternative. The questionnaire also took into account whether the respondent, at the time of the survey or previously, had a transgender experience. Further, the respondents were able to categorize themselves into several variations of sexual identities. In total, the questionnaire was responsive, and the respondents were able to omit questions that were not relevant to them; those with no sexual onset with a partner answered a total of 43 questions while others could be asked a total of 135 questions. Before the first questionnaire was sent out, a letter if introduction was sent, explaining aim, confidentiality and how the results should be used by the Public Health Agency of Sweden. At the same time, a letter was sent out to the guardians of persons under 18 years of age which included contact information to the Swedish Public Health Agency of Sweden, for those who wanted to ask questions. The questionnaires were sent by post to the respondent’s home address. The respondents could choose to fill out the questionnaire on paper or on the web. All respondents received login information in order to be able to log in via Statistics Sweden’s website and complete the form. In total, 67% responded on paper, while 33% responded online.

### Measures

Our hypothesis was that feeling able, i.e. ability, to suggest and thus start to negotiate safer sex is one aspect of life that young people may consider when self-assessing how in control they feel. In order to fulfil our aim, we used the outcome variable “I felt that I could suggest and use a condom or other contraceptive if I wanted to”, with the alternatives, “Yes”, “No” or “I don’t know”. The outcome variables were dichotomized into one (yes) and zero (No and I don’t know.) The outcome variable was analysed in relation to the exposure variable “I have control over my life” with the alternatives “Completely agree”, “Completely disagree” and “Unsure”. The exposure variable was grouped as one (agree) and zero (disagree and unsure). The outcome and exposure variables were chosen based on the hypothesis that they capture the association between control over one’s life situation and the ability for safer sex. To explore how control over one’s life situation and the ability for safer sex intersect with social determinants, the social groups of gender, transgender experience, sexual identity, economy, being foreign-born and social welfare recipiency were chosen. These groups were chosen as they have been shown to impact the ability for safer sex in previous research and constitute key populations in SRHR-policy and research [3, 14, 19, 20, 50, 51, 60–64]. Generally, among those 7755, respondents who filled out the questionnaire answered all questions and the internal item non-responses were relatively low. For the questionnaire item “I have control over my life” the internal non-responses were 1,9% and for questionnaire item “I felt that I could suggest a condom or other protection if I wanted to” 1,8%.

The social groups in the survey were defined as: 1) gender, based on “What is your sex?” with the alternatives “female” “male” or “non-binary gender”, 2) transgender experience, based on“Are you or have you been a transgender person?” with the alternatives “yes” or “no”, 3) sexual identity, based on:“Do you consider yourself currently to be: …with the alternatives “bisexual”, “heterosexual”, “homosexual”, “I do not usually categorise myself sexually” or “other”, 4) economy, based on “How would you describe your household finances?” with the alternatives” very good”,” quite good”, “not particularly good”, “not good at all” or “I don’t know”, 5) foreign-born, based on “country of birth” (register variable defined into regions and continents), and, 6) social welfare recipiency, based on “Receiving social welfare” (register variable based on the sources of household income in 2013 in Sweden), with the alternatives “yes” or “no”.

### Statistical analysis

Descriptive statistics were used to explore and present an overview of the respondents’ answers on both the outcome variable (ability to suggest safer sex) and the exposure variable (control over one’s life). In the descriptive statistics, the variables were graded with *p*-value defined as ***p < 0.001, **p < 0.01, and *p < 0.05, based on the Chi-square test. To further explore the material, and to adjust for the interplay of the six social groups: gender, transgender experience, sexual identity, economy, foreign born and social welfare recipiency, a multivariate logistic regression with adjustment was performed. This was followed by an in-depth intersecting multivariate analysis exploring the intersection of gender and sexual identity using the reference category man and heterosexual. The regression models’ odds ratios (ORs) and adjusted odds ratios (AORs) are presented with 95% confidence intervals throughout. The statistical analysis was conducted in STATA, version 15 (StataCorp LLC, College Station, TX).

### Ethics

An introductory letter was sent by post to the sample group, which explained research ethics and provided information that participation was voluntary. The information stated that informed consent would be assumed if the individual submitted the questionnaire. The letter also informed the recipients that the following background variables would be added to each respondent’s response profile from a number of national registers: sex, being born abroad, income and social welfare recipiency. These data were linked to the response profiles using the personal ID-number that all Swedish residents have. However, as soon as this linking procedure was complete, the personal ID-number was replaced with a dataset ID-code specific to the survey. After this, all possibilities of tracing the data back to the individual’s ID-number were irreversibly blocked, which is a standard procedure for Statistics Sweden. The introductory letter informed the respondents that no personal identification information would be revealed. The questionnaire and the study design were examined and approved by the Regional Ethical Review Board in Stockholm (ref. no.: 2015/5:4). All methods were performed in accordance with the relevant guidelines and regulations from the Ethical Review Board in Stockholm.

## Results

### Control over one’s life

Within the social group based on gender, a higher proportion of young men had control over their life (70%) compared to young women (63%) or young non-binary gender persons (41%). In the social group based on sexual identity a lower proportion of bisexuals (43%) reported that they had control over their life compared to I don’t want to categorize myself sexually (55%) or homosexuals (56%) and heterosexuals (70%). Within the social group of young people with transgender experience, a lower proportion (30%) had control over their lives than was the case among cis-persons (67%). In the social group based on economy, those with not good or insufficient economy reported lower proportions (42%) with control over their lives than those with good or very good economy (71%). In the social group based on birth country, those born outside Sweden reported lower proportions (64%) with control over their lives than those born in Sweden (67%). Within the social group based on social welfare recipiency, lower proportions (42%) reported control over their lives than those with no social welfare recipiency (67%) (Table 1).

**Table 1:** Having control over one’s life. Descriptive statistics for the variable control over one’s life

Social group	Agree		Disagree		Unsure	
	n	% (CI)	n	%* (CI)	n	% (CI)
Gender*** ^**a**^ (*n* = 7459)						
Male	1959	70 [68.3–72.1]	272	12 [10.3–13.1]	458	18 [16.5–19.8]
Female	3035	63 [60.9–64.2]	559	13 [12.2–14.5]	1090	24 [22.7–25.6]
Non-binary gender	30	41 [29.7–53.6]	25	25 [16.6–36.2]	31	34 [23.6–45.5]
Transgender experience*** ^b^ (*n* = 7415)						
Yes	22	30 [18.9–44.5]	25	41 [27.6–54.7]	22	29 [18.8–42.8]
No	4978	67 [65.3–67.9]	825	12 [11.4–13.3]	1543	21 [20.0–22.2]
Sexual identity*** ^**c**^ **(***n* = 7109)						
Heterosexual	4411	70 [68.6–71.3]	581	11 [9.7–11.6]	1206	20 [18.4–20.7]
Bisexual	184	43 [37.8–48.9]	105	26 [21.2–31.2]	134	31 [26.0–36.1]
Homosexual	73	56 [42.2–61.0]	26	23 [15.6–32.6]	37	25 [18.1–34.2]
I don’t usually categorize myself sexually	197	55 [49.0–61.3]	63	17 [13.2–22.2]	92	28 [22.2–33.5]
Economy*** ^**d**^ (*n* = 7224)						
Very good or sufficient	4481	71 [70.0–72.6]	558	10 [8.6–10.3]	1225	19 [18.2–20.5]
Not very good or insufficient	418	42 [38.4–45.6]	254	28 [25–31.9]	288	30 [26.4–33.2]
**Birth country/region** ^**e**^ (*n* = 7459)						
Born in Sweden	4571	67 [65.3–67.9]	761	13 [11.5–13.5]	1421	21 [19.8–22.0]
Born outside Sweden	453	64 [59.7–67.5]	95	13 [10.8–16.3]	158	23 [19.7–26.6]
Social welfare recipiency^*** f^ (*n* = 7341)						
No	4892	67 [65.7–68.2]	806	12 [11.3–13.1]	1517	21 [19.8–22.0]
Yes	51	42 [32.8–51.3]	36	27 [19.8–36.3]	39	31 [22.9–40.2]

***p < 0.001, **p < 0.01, *p < 0.05

^a^Gender: Self-reported variable

^b^Transgender experience: Self-reported variable

^c^Sexual identity: Self-reported variable. The five alternatives: heterosexual, homosexual, bisexual, I don’t usually categorize myself sexually were limited to four, since those who answered: I don’t know and other were removed from the analysis

^d^Economy: Self-reported variable. The alternatives: very good, fairly good, not very good, or not good at all were grouped into two categories

^e^Foreign-born: Register variable from Statistics Sweden. The categories for birth country and birth region follow Statistics Sweden’s alternatives: Sweden, the Nordic countries except Sweden, Europe except the Nordic countries, Africa, Asia, North America, South America

^f^Social welfare recipiency: Register variable from Statistics Sweden

### Ability to suggest safer sex

Within the social group based on gender, a slightly higher proportion of young women (90%) had ability to suggest safer sex compared to young men (89%) or young non-binary gender persons (78%). In the social group based on sexual identity, a lower proportion of homosexuals (78%) reported that they had ability to suggest safer sex compared to I don’t want to categorize myself sexually (83%) or bisexuals (84%) and heterosexuals (90%). Within the social group of young people with transgender experience, a lower proportion (76%) had ability to suggest safer sex than was the case among cis-gendered young people (89%). In the social group based on economy, those with not good or insufficient economy reported lower proportions (85%) with ability to suggest safer sex than those with good or very good economy (90%). In the social group based on birth country those born outside Sweden reported lower proportions (83%) with ability to suggest safer sex than those born in Sweden (90%). Within the social group based on social welfare recipiency lower proportions (82%) reported ability to suggest safer sex than those with no social welfare recipiency (90%) (Table 2).

**Table 2:** Ability to suggest safer sex. Descriptive statistics for the variable ability to suggest safer sex

Social group	Yes		No		Can’t /don’t want to answer	
	n	% (CI)	n	%* (CI)	n	% (CI)
**Gender** ^**a**^ (*n* = 5719)						
Male	1717	89 [87.4–90.6]	87	5 [4.3–6.7]	96	6 [4.5–6.8]
Female	3473	90 [88.4–90.9]	172	5 [4.0–5.7]	174	5 [4.7–6.6]
Non-binary gender	45	78 [61.8–88.1]	2	5 [0.9–25.3]	10	17 [8.5–31.1]
**Transgender experience** ^b^ (*n* = 5744)						
Yes	43	76 [56.8–88.0]	6	10 [3.0–29.6]	6	14 [5.5–32.1]
No	5165	89 [88.4–90.4]	255	5 [4.3–5.7]	272	6 [4.9–6.4]
Sexual identity*** ^**c**^ **(***n* = 5599)						
Heterosexual	4510	90 [89.7–91.7]	199	5 [3.9–5.3]	190	5 [4.0–5.5]
Bisexual	300	84 [78.0–88.1]	27	9 [6.2–14.4]	23	7 [4.1–11.2]
Homosexual	72	78 [68.6–84.8]	5	4 [1.5–9.7]	26	18 [12.0–26.9]
I don’t usually categorize myself sexually	210	83 [76.2–88.3]	18	6 [3.3–10.0]	19	11 [6.8–17.8]
Economy*** ^**d**^ (*n* = 5657)						
Very good or sufficient	4438	90 [89.3–91.4]	204	5 [4.1–5.6]	204	5 [4.2–5.7]
Not very good or insufficient	700	85 [82.2–88.1]	51	6 [4.6–8.8]	60	8 [6.2–10.8]
Birth country/region*** ^**e**^ (*n* = 5776)						
Born in Sweden	4823	90 [89.4–91.3]	31	8 [5.5–11.2]	37	9 [6.7,12.9]
Born outside Sweden	412	83 [78.5–86.3]	230	5 [4.0–5.3]	243	5 [4.4,5.8]
Social welfare recipiency* ^**f**^ (*n* = 5692)						
No	5070	90 [88.8–90.7]	247	5 [4.3–5.7]	259	5 [4.6–6.0]
Yes	94	82 [72.6–88.0]	10	7 [3.6–14.3]	12	11 [6.3–19.1]

***p < 0.001, **p < 0.01, *p < 0.05

^a^Gender: Self-reported variable

^b^Transgender experience: Self-reported variable

^c^Sexual identity: Self-reported variable. The five alternatives: heterosexual, homosexual, bisexual, I don’t usually categorize myself sexually were limited to four, since those who answered: I don’t know and other were removed from the analysis

^d^Economy: Self-reported variable. The alternatives: very good, fairly good, not very good, or not good at all were grouped into two categories

^e^Foreign-born: Register variable from Statistics Sweden. The categories for birth country and birth region follow Statistics Sweden’s alternatives: Sweden, the Nordic countries except Sweden, Europe except the Nordic countries, Africa, Asia, North America, South America

^f^Social welfare recipiency: Register variable from Statistics Sweden

### Association analysis

The results from the univariate logistic model show an association between having control over one’s life and ability for safer sex, with higher odds for safer sex (OR 1.7, *p* value < 0.000) among those who stated that they had control over their lives compared to those who did not. The results from the adjusted multivariate logistic regression model show that the association between having control over one’s life and ability for safer sex remains (1.45 AOR, *p*-value 0.003) after controlling for the social determinants: gender, transgender experience, sexual identity, economy, being foreign-born and receiving social welfare (Table 3).

**Table 3:** Multivariate logistic regression exploring how control over one’s life affects the ability for safer sex

Exposure/Social groups		Univariate model Outcome variable: Ability to suggest safer sex			Multivariate model Outcome variable: Ability to suggest safer sex	
	OR ^f^	95% CI ^g^	***p***-value	AOR ^r^	95% CI ^g^	***p***-value
**Control over one’s life** ^**a**^						
No (*n* = 2244)	1	ref.	ref.	1	ref.	ref.
Yes (*n* = 4865)	1.676848	1.356296 - 2.073161	0.000	1.459745	1.135624–1.876373	0.003
**Gender**						
Male (*n* = 2648)	1	ref.	ref.	1	ref.	ref.
Non-binary gender (*n* = 54)	0.5084281	0.2287345–1.130127	0.097	0.5881884	0.2394457–1.44486	0.247
Female (*n* = 4546)	1.111739	0.8964234–1.378771	0.335	1.329467	1.062337–1.663767	0.013
**Transgender experience**						
No (*n* = 7167)	1	ref.	ref.	1	ref.	ref.
Yes (*n* = 44)	0.4391327	0.1707182 - 1.129566	0.088	1.46532	0.4280708–5.015905	0.543
**Sexual identity** ^**b**^						
Hetero (*n* = 6315)	1	ref.	ref.	1	ref.	ref.
Bi(*n* = 433)	0.569767	0.3813934 - 0.8511799	0.006	0.6351049	0.4168464–0.9676425	0.035
Homo (*n* = 140)	0.3753941	0.2283796 - 0.6170461	0.000	0.3517219	0.2075508 - 0.5960388	0.000
I don’t usually categorize myself sexually (*n* = 360)	0.527728	0.3352254 - 0.8307749	0.006	0.5724697	0.3556961–0.9213527	0.022
**Economy** ^**c**^						
Very good or sufficient (*n* = 6115)	1	ref.	ref.	1	ref.	ref.
Not very good or insufficient (*n* = 919)	0.6967761	0.5231338 - 0.9280549	0.014	0.8881435	0.6551841 - 1.203935	0.445
**Foreign-born** ^**d**^						
Born in Sweden (*n* = 6602)	1	ref.	ref.	1	ref.	ref.
Born outside Sweden (*n* = 646)	0.5170427	0.3831206–0.6977781	0.000	0.6471391	0.4493218–0.9320469	0.019
**Social welfare recipiency** ^**e**^						
No social welfare (*n* = 7024)	1	ref.	ref.	1	ref.	ref.
Received social welfare (*n* = 119)	0.5321847	0.3154711–0.8977703	0.018	0.6321254	0.3487383 - 1.145795	0.131

Exposure:

^a^ Control over one’s life: Self-reported variable. The three alternatives were grouped into two: 1) “Yes”, and 0) “No” or “I am not sure”

Social determinants:

^b^ Sexual identity: Self-reported variable. The five alternatives: heterosexual, homosexual, bisexual, I don’t usually categorize myself sexually were limited to four, since those who answered with I don’t know and “other” were removed from the analysis

^c^ Economy: Self-reported variable. The four alternatives very good, fairly good, not very good, and, not good at all, were grouped into two categories

^d^ Foreign-born: The categories for birth country and birth region follow Statistics Sweden’s alternatives: Sweden, the Nordic countries except Sweden, Europe except the Nordic countries, Africa, Asia, North America, South America, Oceania, Other, and were grouped into two categories

^e^ Social welfare recipiency: Register variable drawn from Statistics Sweden (SCB)

Statistics:

^f^ Likelihood: OR: odds ratio (model 2), AOR: adjusted odds ratio (model3), CI: 95% confidence interval

^g^ Proportions: % are weighted proportions according to UngKAB15 to ensure that the sample group responses are representative of the total population aged 16–29 in Sweden

### Intersections of gender and sexual identity

The results from the intersecting multivariate logistic regression model analysis (Table 4) are based on the 12 intersecting social positions, with gender being crosscut with sexual identity (Table 4). The model gives men, women and non-binary gender persons four positions each defined on the basis of their sexual identity. The model did not show significant results, and as the groups are small the confidence interval is broad. However, the model shows a pattern. For men, the position of being a man and not usually categorizing oneself sexually was associated with lower odds (OR 0.58) for the ability to suggest safer sex compared to the position of being a man and heterosexual (reference category). For women, the positions associated with lower odds for the ability to suggest safer sex were found in being a woman and homosexual (OR 0.19), a woman and bisexual (OR 0.60) or a woman and not usually categorizing oneself sexually (OR 0.77) compared to the position of being a man and heterosexual (reference category). For non-binary gendered persons, the positions associated with lower odds for the ability to suggest safer sex were found in being non-binary gender and heterosexual (OR 0.59), non-binary gender and bisexual (OR 0.45), and also non-binary gender and not usually categorizing oneself sexually (OR 0.52).

**Table 4:** Intersecting multivariate logistic regression exploring how control over one’s life affects the ability for safer sex based on the intersections between gender and sexual identity

	Univariate model Ability to suggest safer sex		
	OR ^**b**^	95% CI ^c^	***p***-value
**Control over one’s life** ^**a**^			
No, I had no ability to suggest safer sex at last sex (*n* = 2244)	1	ref.	ref.
Yes, I had ability to suggest safer sex at last sex (*n* = 4865)	1.584825	1.263933–1.987185	0.000
Gender * heterosexual identity			
Male and heterosexual (*n* = 2406)	1	ref.	ref.
Female and heterosexual (*n* = 3890)	1.61278	1.276727–2.037288	0.000
Non-binary and heterosexual (*n* = 19)	0.599831	0.2120385–2.298937	0.555
Gender * homosexual identity			
Male and homosexual (*n* = 54)	1.891751	0.5623386–6.364	0.303
Female and homosexual (*n* = 83)	0.1930998	0.1080428–0.345118	0.000
Non-binary and homosexual (*n* = 3)	–	–	–
Gender * bisexual identity			
Male and bisexual (*n* = 81)	1.700775	0.6344579–4.559221	0.291
Female and bisexual (*n* = 334)	0.6083382	0.3952046–0.9364146	0.024
Non-binary and bisexual (*n* = 18)	0.4529464	0.1453062–1.411919	0.172
Gender * open sexual identity			
Male and I don’t usually categorize myself sexually (*n* = 107)	0.581003	0.2519825–1.339635	0.203
Female and I don’t usually categorize myself sexually (*n* = 239)	0.774941	0.4494872 - 1.336042	0.359
Non-binary and I don’t usually categorize myself sexually (*n* = 14)	0.5292683	0.0856194 - 3.271748	0.494

Exposure:

^a^ Control over one’s life: Self-reported variable. The three response alternatives were grouped into two: 1) “Yes”, and 0) “No” or “I am not sure”

Statistics:

^b^ Likelihood: OR: odds ratio (model 4), CI: 95% confidence interval

^c^ Proportions: % are weighted proportions according to UngKAB15 to ensure that the sample group responses are representative of the total population aged 16–29 in Sweden

The positions with higher odds for the ability to suggest safer sex among men were being a man and homosexual (OR 1.89), and being a man and bisexual (OR 1.70). For women, the position with higher odds for the ability to suggest safer sex was being a woman and heterosexual (OR 1.61). The position of being of non-binary gender was not associated with higher odds for the ability to suggest safer sex in any position compared to the reference category, being a man and heterosexual.

## Discussion

The overarching aim of this paper was to explore the association between control over one’s life and the ability to suggest safer sex among young people in Sweden. Moreover, we wanted to explore how this plays out in social groups based on gender, transgender experience, sexual identity, economy, being foreign-born and social welfare recipiency, followed by an in-depth analysis of the intersection of gender and sexual identity. The results illustrate that there is an association between having control over one’s life and the ability to suggest safer sex among young people. Also, our results indicate that the ability to suggest safer sex is dependent on the intersection of gender and sexual identity. In the following section, this will be discussed in relation to existing literature as its implication for policy and practice.

### Control over life a resource for ability to suggest safer sex

The results in the multivariate logistic regression illustrate that there is an association between having control over one’s life and the ability to suggest safer sex among young people. This indicates that feeling able to suggest, and maybe start to negotiate on safer sex, is one aspect of life associated with the self-reported factor of how in control, over their own life young people feel they are. Based on these findings we suggest that that having control over one’s life is a resource for attaining sexual health and can be understood as a determinant of the ability to suggest safer sex. Having control over one’s life can be seen as part of what the WHO states as “Social inclusion and non-discrimination” and “Structural conflict” and through that part of the social determinants of health [12]. These results contribute to the field of SRHR by addressing the broader social context in which young people perceive their social lives relative to others. This is in line with the comprehensive understanding of SRHR in the Guttmacher-Lancet commission [2] and with previous research indicating that the individual risk perspective cannot account for all explanations of safer sex [47, 48, 65, 66]. The results might also help to overcome the dominant individual risk perspective, which has for decades constituted the overarching focus of analyses of safer sex among young people [19]. Our results point to that young people’s resources for sexual health via control over their lives and the ability to suggest safer sex instead needs a closer link to ideas of social inclusion and health promotion [12, 16]. Our findings underscores previous research which has shown that social determinants are crucial to SRHR [20, 67] and that the resources for sexual health are found in social life [16, 28, 68].

### Unequal resources for sexual health

The results also contribute with novel findings to the field by revealing how the social resource, i.e. the determinant control over one’s life plays out in a national sample of young people. The results from the descriptive statistics indicate that control over one’s life follows is unequal distributed where the highest proportions of those with resources (in terms of having control over one’s life) were found among respondents who were male, heterosexual, cis-gendered, had a good economy, were born in Sweden and were not receiving social welfare. In contrast, lower proportions were found among women, non-binary gender persons, people with transgender experiences, sexual minorities, foreign-born young people, and youths with a poor economy or who were receiving social welfare. These findings coincide with previous research focused on gender-based analysis on social determinants linked to transgender experience, sexual minorities, economy, migration and various forms of social welfare recipiency [31, 33, 69]. These unequal resources for control over one’s life and thus ability to suggest safer indicates a need to further improve social conditions for those with less resources. Such improvement would help to adjust for unfair social resources and help to reach the goal of health equity in SRHR [11, 70].

### Intersections and resources for sexual health

Drawing on the evidence that sexual minorities are vulnerable to sexual ill health [2] we explored the intersections of gender and sexual identity and its connection to resources for sexual health. The intersecting multivariate regression analysis illuminate how intersectionality can facilitate an understanding of the complexity of what at first sight is a social group with resources for sexual health. The results from our intersecting multivariate regression model indicates how resources are distributed in a more complex pattern depending on the intersection of gender and sexual identity. Among the majority of the 12 intersecting positions within the social groups (i.e. determinants) defined by gender and sexual identity, the lowest odds for the ability to suggest safer sex were found among the position of being either a woman *and* homosexual. This can be understood in terms of lower or no need for safer sex due to sexual practice with a same sex partner. However, the position with the next lowest results were being non-binary gender *and* bisexual. This aligns with previous research showing that bisexuals are vulnerable to sexual ill health [71]. For men, the results illuminated complexity as the two positions being a man *and* homosexual, or being man *and* bisexual were associated with higher odds than the two positions of being a man *and* heterosexual or a man *and* not usually categorizing oneself sexually. Altogether the results from the intersecting multivariate regression point to that an intersectional perspective illuminates vulnerable groups with less resources for health [43]. Our results suggest that intersectional models provide key knowledge concerning how to understand the complexity of resources for the ability to suggest safer sex. Moreover, to our knowledge this is one of few studies based on a total population sample that reveals the complexity of intersections based on gender and four categories of sexual identity in relation to SRHR. Our findings may therefore be helpful to both national policy makers and practitioners.

### Implications for policy and practise

The results suggest a need for policy that encourages practice to consider the way in which gender intersects with sexual identity and creates social positions with privileged and unprivileged conditions with regard to resources linked to the ability for safer sex. This indicates that interventions and programmes need to address the social life situation around young people, and not only be designed on the basis of gender or sexual identity. Giving young people more control over their social lives can improve the preconditions for safer sex and sexual health. Our results place the ability to suggest safer sex and sexual health in the broader context of inclusion and control in social life. However, the formation of an inclusive society that contributes to intersectional resources for the attainment of sexual health and SRHR cannot be left to young people from sexual minorities or viewed as an individual issue relating to the ability for safer sex. Instead, inclusion must stem from a broader social awareness of gender equality and sexual rights. To support young people’s inclusion in social life, their sexual rights must be respected and unequal conditions, social stress and discrimination in social life need to be eliminated [11, 15, 72]. One way to achieve inclusion and non-discrimination is to make knowledge-based platforms available to practitioners or to provide basic education in sexual health and sexual rights in higher education in order to guide practitioners [73, 74]. These efforts could strengthen the intersecting resources for sexual rights, and thus the resources for attaining sexual health among young people. In a national Swedish context, this may also constitute a means of working in a more well-tailored fashion with the public health domain of control and inclusion [11]. Our results suggest that resources for the ability to suggest safer sex vary by sexual identity. A more intersectional understanding of sexual health promotion is therefore needed.

### Suggestions for future research

Our results indicate that social life and safer sex are complex and intertwined with power structures connected to gender and sexual identity. The descriptive findings show that young men in general had more control over their lives than women and non-binary gendered persons. However, both the multivariate and intersecting regression models showed that the position of being a woman *and* heterosexual generated more resources than being a man *and* heterosexual or being a man *and* not usually categorizing oneself sexually. This can be seen as complexities in health equity revealed using the theory and methods of intersectionality [43, 75]. In future research, this complexity of resources needs to be further investigated in order to fully understand the ability to suggest safer sex.

### Methodological reflections

This study includes an analysis of social groups of varying sizes, which means that the confidence intervals are broad. On the one hand, this can be interpreted as producing less valid findings, while on the other the results may be shedding light on the reality of diversity within a total population. Smaller groups are often left out in health surveys, and in some population studies, small groups are not included at all in the analysis and presentation of results [76]. This might contribute to making health inequalities less visible and may even add to vulnerability and marginalisation. This paper strives to make intersecting positions visible, for which reason it was important to include smaller groups both in the descriptive statistics and in the regression models. This research strategy has been highlighted by the UN [77] as a means of further developing national monitoring and global reporting in the field of SRHR. Since social life is in constant change, we need to be aware about the time and context of our definitions of both social groups and positions, and also our research findings [78]. This means that the social groups examined, and their internal social positions, are relevant to the Swedish context, and may be transferable to countries similar to Sweden. It would be of interest to learn more about similarities and differences between contexts on the basis of a more widespread use of intersectional perspectives in the research on safer sex. From the non-response analysis we know that foreign-born persons and young people from homes with a lower socioeconomic position did not answer the questionnaire to the same extent as others. Even though the study design included design weights to calibrate for this, the non-response may still be reflected in the data.

## Conclusion

Young people’s control over their life situation can be defined as a social resource for the ability for safer sex, sexual health and SRHR for young men, women and non-binary gendered persons. However, gender cannot account for all differences in resources for sexual health and needs to be complemented with an intersectional perspective, and thus transformed into an intersectional resource gradient for safer sex. Implications for policy and practitioners would involve addressing and improving gender equality and sexual rights relating to young people from sexual minorities, and tailoring interventions in a way that takes the intersections between gender and sexual identity into consideration.

## Data Availability

The data that support the findings of this study belongs to The Public Health Agency of Sweden, and restrictions apply to the availability of these data, which were used under license for the current study. The analyses are available from the authors upon reasonable request and with permission of The Public Health Agency of Sweden. For further questions on availability of data and materials, please contact the corresponding author.
